# Factors Affecting Preventive Treatment Outcomes for Patients With Newly Diagnosed Chronic Migraine and Their Compliance With Treatment Recommendations in Chongqing Province, China: An Open-Label Prospective Study With Retrospective Baseline

**DOI:** 10.3389/fneur.2020.00227

**Published:** 2020-04-09

**Authors:** Dongli Yuan, Yixin Zhang, Qin Li, Yuhua Lv, Xuelian Li, Yichuan Yu, Wangwen Li, Ge Tan

**Affiliations:** ^1^Department of Intelligent Medical Systems, Institute of Medical Information, Chongqing Medical University, Chongqing, China; ^2^Department of Neurology, The First Affiliated Hospital of Chongqing Medical University, Chongqing, China; ^3^Department of Neurology, Chongqing Zhongshan Hospital, Chongqing, China; ^4^Department of Neurology, Chongqing Banan Hospital, Chongqing, China; ^5^Department of Neurology, Chongqing Hechuan District Hospital, Chongqing, China; ^6^Department of Neurology, Chongqing Yongchuan District Hospital, Chongqing, China; ^7^Department of Neurology, Chongqing Sanxia Center Hospital, Chongqing, China

**Keywords:** chronic migraine, preventive therapy, patient compliance, therapeutic effect, medication

## Abstract

**Objective:** This study aims to investigate the factors affecting the efficacy of first oral prophylaxis in patients with chronic migraine (CM) and to assess patient compliance with their medication regimens.

**Method:** To identify the therapeutic effect of prevention medication in 740 patients with newly diagnosed CM that did not receive any preventive treatments after 4 weeks in an open-label prospective study with retrospective baseline from January 2016 to January 2018, the factors that may affect the outcomes of preventive treatment were analyzed based on the demographic characteristics, migraine characteristics, family history of headache, and history of medication overuse. Moreover, the patients were followed up to evaluate their compliance with and the side effects of the medication at 4 weeks and at 12 weeks.

**Results:** After 4 weeks of prophylaxis, 94.3% (*n* = 698) of the patients persisted with taking the medicine. The treatment was effective for 61.7% of CM patients (*n* = 431) and ineffective for 38.3% (*n* = 267). The results showed that the effectiveness of the preventive treatment was related to the number of headaches per month, and the effect was better for patients with headaches for 15–20 days/month than for those with headaches for 26–30 days/month (OR = 2.78, 95% CI: 1.26–5.75, *P* = 0.006). After 12 weeks of treatment, only 34.5% (*n* = 255) of the patients persisted with taking the medicine. The most common reason for non-compliance in CM patients is appointment difficulty in a headache clinic (31.8%).

**Conclusion:** The effect of CM prophylaxis was related to the frequency of headache. Only 34.5% of the patients continued to take medicine after 12 weeks of treatment, suggesting that patient compliance needs to be enhanced in the prophylaxis of CM. For the Chinese headache society, the best way to increase patient compliance should be treatment at dedicated headache centers and timely visits to headache specialists.

## Introduction

Chronic migraine (CM) is a common disabling disease with a global prevalence of ~1–2% (1, 2). The disease seriously affects the quality of life of patients and wastes numerous social resources (3). At present, CM treatment is still mostly a drug-based treatment, including acute treatment (4) and preventive treatment. Unfortunately, patients often respond poorly to preventive treatment (5–7) and have a low compliance with long-term medications (8). The purpose of this study was to determine the factors that affect the efficacy of first-time treatment in patients with CM and to assess patient compliance with oral prophylactics.

## Methods

### General Information

Data of CM patients who went to the specialized headache clinics of six hospitals (including The First Affiliated Hospital of Chongqing Medical University, Chongqing Zhongshan Hospital, Chongqing Banan Hospital, Chongqing Hechuan District Hospital, Chongqing Yongchuan District Hospital, and Chongqing Sanxia Center Hospital) in Chongqing, China, from January 2016 to January 2018 were collected. The CM patients were diagnosed according to the criteria of the International Headache Disease Classification 3rd Edition (ICHD-3 beta) (9). The patient's demographic characteristics, headache history, headache characteristics, family history of headache, and medication overuse (ICHD-3 beta) were recorded at the time of the first visit. All of the patients were required to record headache diaries from the enrollment. During the course, the patients were followed up once every 4 weeks at the headache clinic. Compliance and medication status were collected in the form of questionnaires. If the patients could not come to the clinic, we would follow up by telephone or WeChat (WeChat is a mobile text and voice messaging communication service which is the most widely used in China). It is an open-label prospective study with a retrospective baseline.

#### Inclusion Criteria

Patients 18 years of age or older with headaches that were diagnosed as CM were included in the study. We recruited prophylaxis-naïve patients with newly diagnosed CM that did not receive any preventive treatments including pharmacological (beta-blockers, antidepressants, ant-epileptic drugs, or calcium channel blockers), botulinum toxin injection, or acupuncture therapy before participating in this study.

#### Exclusion Criteria

The exclusion criteria were as follows: (i) patients who were younger than 18 years of age, (ii) headache type other than CM, (iii) previous history of migraine prophylaxis before enrollment, (iv) pregnancy or nursing status, (v) hepatic or renal disorder, nephrolithiasis, or other severe systemic diseases, and (vi) incomplete medical records.

After receiving detailed information about the adverse reactions of each drug, the patients who met the study criteria were given at least one of the following preventive drugs under the guidance of a headache expert: metoprolol (25–50 mg/day), flunarizine (5–10 mg/day), amitriptyline (25–50 mg/day), and topiramate tablets (75–100 mg/day); all patients' medications were gradually increased from a small dose to the maximum dose within 2 weeks. Furthermore, all patients with medication overuse stopped using painkillers that were previously used before starting the prophylactic treatment.

### Method

To analyze the effect of preventive treatment on CM patients, the effective treatment aimed to reduce the number of headache days by more than or equal to 50% after 4 weeks of medication, and the clinical characteristics of the patients were analyzed to identify the factors that affected the preventive treatment outcomes. Patients with CM were followed up to evaluate their compliance with the medication regimen after 4 and 12 weeks of treatment. Headache intensity was assessed by a 10-point visual analog scale (VAS) (0, no pain; 10, severe pain prohibiting daily activities). The side effects experienced by the patients under treatment were recorded, and the reasons for non-compliance were also recorded. The primary endpoints are medication compliance, side effects of the drugs, and the reasons for non-compliance. The secondary endpoints are general information of CM patients and headache-related factors of CM patients.

### Statistical Processing

The statistical analysis was conducted using Statistical Package for the Social Sciences (SPSS), version 20.0 (Chicago, IL, USA). All normally distributed measurement data were expressed as x ± s, and *t*-tests were used for comparisons between groups. The count data are expressed as a ratio, and the χ^2^-test was used for comparisons between groups. A multivariate regression analysis model was used to estimate the odds ratios (ORs) of age, sex, CM history, duration of headache attack, headache days per month, and VAS as well as their 95% confidence intervals (CIs). Considering that preventive outcomes were clinically distinct entities that associated with individual risk factors, we chose potential confounding factors that would be relevant for outcomes. Thus, age and sex were adjusted in the multivariate model. A two-sided *P* < 0.05 was considered as statistically significant.

## Results

### Therapeutic Effect and Medication Compliance

A total of 740 patients met the study criteria, and 698 patients continued to take medicine after 4 weeks, including 156 males and 542 females; 387 patients (55.4%) had a family history of headache, and 468 patients (67.0%) had a history of drug overuse. All the patients received prophylactic medication: 65 patients took metoprolol, 351 patients took flunarizine, 167 patients took amitriptyline, 62 patients took topiramate tablets, and 53 patients took metoprolol and flunarizine at the same time. The efficacy of the medication for the patient was evaluated after 4 weeks of prophylactic treatment; the medication was effective for 431 patients (61.7%) and ineffective for 267 patients (38.3%). Metoprolol was effective for 51 patients (78.5%); flunarizine was effective for 233 patients (66.4%); amitriptyline was effective for 57 patients (34.1%); topiramate was effective for 47 patients (75.8%); metoprolol and flunarizine, used in combination, were effective for 43 patients (81.1%). At the 4-week follow-up, 698 patients continued to take the medicine, and the compliance rate was 94.3%. At the 12-week follow-up, only 255 patients continued to take the medicine, and the medication compliance rate was 34.5%.

### Factors Affecting Treatment Outcomes

The CM history in the effective-treatment group was shorter than that in the ineffective-treatment group (*P* = 0.001). The patients in the effective-treatment group had fewer headache days per month than those in the ineffective-treatment group (*P* = 0.013). The duration of each headache episode was longer in the ineffective-treatment group than in the effective-treatment group (*P* = 0.038). There were no significant differences in age, sex, body mass index (BMI), education background, age of headache and CM onset, headache history, duration of headache attack, VAS, migraine characteristics, symptoms associated with headache, family history of headache, or medication overuse between patients in the effective-treatment group and patients in the ineffective-treatment group (*P* > 0.05) (shown in Tables 1, 2). After adjusting for age and sex in the multivariate regression analysis, the prevention effect was related to the number of headaches per month (OR = 2.78, 95% CI: 1.26–5.75, *P* = 0.006). There were no significant differences in the CM history, duration of headache attack, or VAS (*P* > 0.05) between the two groups. The therapeutic effect could not be predicted (shown in Table 3).

**Table 1:** General information of chronic migraine patients after the first oral prophylaxis treatment.

	**Ineffective** **group (*n* = 267)**	**Effective** **group (*n* = 431)**	***P***
Age (years)	47.2 ± 10.5	45.7 ± 11.3	0.578
Body mass index (kg/m^2^)	24.4 ± 3.9	23.4 ± 3.2	0.386
Sex [*n* (%)]			0.365
Male	64 (24.0)	92 (21.3)	
Female	203 (76.0)	339 (78.7)	
Educational background [*n* (%)]			0.427
Below high school	203 (76.0)	348 (80.7)	
High school and above	64 (24.0)	83 (19.3)	
Medication overuse [*n* (%)]	172 (64.4)	296 (68.7)	0.525
Family history of headache [*n* (%)]			0.761
Yes	148 (55.4)	239 (55.5)	
No	119 (44.6)	192 (44.5)	

**Table 2:** Headache-related factors of chronic migraine (CM) patients after the first oral prophylaxis treatment.

	**Ineffective** **group (*n* = 267)**	**Effective** **group (*n* = 431)**	***P***
Age of headache onset (years)	32.3 ± 10.9	34.5 ± 12.3	0.682
Headache history (years)	19.8 ± 10.7	17.4 ± 11.6	0.629
Age of CM onset (years)	38.6 ± 11.8	40.7 ± 10.2	0.587
CM history (years)	10.6 ± 7.2	7.1 ± 6.1	0.001
Duration of headache attack (h)	16.5 ± 9.6	13.2 ± 11.2	0.038
Visual analog scale	7.3 ± 2.1	7.5 ± 1.9	0.634
Headache days per month (days)	24.1 ± 5.5	21.2 ± 6.4	0.013
15–20 days [*n* (%)]	65 (24.3)	205 (47.6)	
21–25 days [*n* (%)]	21 (7.9)	34 (7.9)	
26–30 days [*n* (%)]	181 (67.8)	192 (44.5)	
Migraine characteristics [*n* (%)]			
Unilateral	82 (30.7)	107 (24.8)	0.137
Pulsatile	137 (51.3)	268 (62.2)	0.629
Aggravation by routine physical activity	185 (69.3)	331 (76.8)	0.943
Symptoms associated with headache [*n* (%)]			
Nausea	194 (72.7)	320 (74.2)	0.612
Vomiting	112 (41.9)	185 (42.9)	0.754
Photophobia and phonophobia	125 (46.8)	193 (44.8)	0.631

**Table 3:** Multivariate regression analysis of the influence on the curative effect of chronic migraine (CM).

	**OR**	**95% CI**	***P***
Age	1.06	0.89–1.11	0.698
Sex	1.46	0.72–2.88	0.481
CM history	0.92	0.86–1.06	0.212
Duration of headache attack	0.88	0.90–1.05	0.113
Headache days per month	2.78	1.26–5.75	0.006
Visual analog scale	1.21	0.81–1.55	0.686

### Side Effects and the Reasons for Non-compliance

According to our follow-ups, a total of 104 patients (14.1%) developed side effects, including 3 patients with metoprolol, 52 patients with flunarizine, 29 patients with amitriptyline, 8 patients with topiramate, and 12 patients with metoprolol and flunarizine combination. The most common side effects are dry mouth, drowsiness, and gastrointestinal reactions. The reasons for non-compliance among 485 patients are side effects, unsatisfied effect, forgot to take the medicine, appointment difficulty in a headache clinic, and pain relief (shown in Table 4). The most common reason is appointment difficulty in a headache clinic (31.8%).

**Table 4:** The reasons for non-compliance of chronic migraine (CM) patients with oral prophylactic medication.

	**CM patients with non-compliance (*n* = 485)**
Side effects [*n* (%)]	56 (11.5)
Unsatisfied effect [*n* (%)]	145 (29.9)
Forget to take medicine [*n* (%)]	46 (9.5)
Appointment difficulty in headache clinic [*n* (%)]	154 (31.8)
Pain relief [*n* (%)]	32 (6.6)
Some reasons unknown [*n* (%)]	52 (10.7)

## Discussion

CM has a higher severe disability rate than episodic migraine, and the efficacy of preventive treatment and the treatment compliance rates are poor (2, 8). This study analyzed the baseline clinical characteristics that may affect the effectiveness of first-time prophylaxis and assessed patient compliance with the prophylaxis regimen.

Previous observational studies have shown that, for patients with CM who receive prophylactic treatment, the medication compliance rate was 65.3–92.4% after 4 weeks of follow-up, 30.9–76.0% after 12 weeks, 21–80% after 6 months, and 31–56% after 12 months (10, 11). Moreover, Hepp et al. also conducted a 4-year retrospective study in the USA that evaluated 8,707 patients with CM who received first-time preventive treatments including topiramate tablets, flunarizine, and propranolol (12). The authors found that, regardless of whether the medication was used as the first preventive treatment or as a new preventive treatment after the medication was changed, patient compliance gradually decreased as the medication time was extended, and the drug was discontinued within 2–3 months on average. Approximately 45% of patients chose to stop the first preventive treatment after 4 weeks of taking the medicine, and the compliance rate showed a significant linear downward trend. These findings are consistent with the results of this study. This study found that the patient compliance rate was 94.3% at the 4-week follow-up and 34.5% at the 12-week follow-up. The patient compliance rate also showed a significant decrease with a more prolonged medication time.

The domestic and international guidelines for CM prevention and treatment recommend that the observation period for preventive treatment should be at least 1–6 months, and the effects of an effective preventive treatment need to last for ~6 months (13, 14). Some studies have suggested that for patients with chronic diseases, a medication compliance rate below 80% may be related to a poor prognosis of the disease treatment (15, 16). At present, this relationship is uncertain, and there is little research on which factors affect patient medication compliance. Studies have shown that the adverse reactions of the preventive drugs and the patients' lack of confidence in drugs and in drug treatment are the main reasons behind the discontinuation of medications (8, 12, 17). In this study, the effective rate of the first preventive treatment after 4 weeks was 61.7%; the therapeutic effect may affect patient medication compliance, so it is valuable to identify the factors that influence the efficacy of preventive treatment for CM patients.

The univariate analysis in this study suggests that the CM history, duration of headache attack, VAS, and the headache days per month may be related to a therapeutic effect. After a multivariate regression analysis, only the headache days per month were statistically significant (*P* < 0.01), which indicates that this factor may be related to the effectiveness of the first preventive treatment. However, the current research results on the relationship between the number of headache days per month and the effectiveness of preventive treatment are still inconclusive. Luconi et al.'s (18) prognostic study on CM showed that the headache days were not related to the effectiveness of the preventive treatment, while the study conducted by Gaul et al. on CM with 1-year follow-ups found that the patients with more headache days had a better therapeutic effect; thus, the results are still inconclusive (19). A newly published systematic review of factors that affect CM prognosis included 27 observational studies and clinical controlled trials on drugs for CM. The results suggest that moderate-quality research evidence indicates that depression/anxiety, drug overuse, poor sleep quality, patient stress, and low Hamilton Depression Rating Scale scores are associated with poor treatment outcomes; low-quality evidence suggests that high expectations for drugs, age, age of CM onset, headache days, headache intensity, BMI, headache disability index score, and current occupational status are potential predictors of prognosis (20). Some of these findings are inconsistent with the results of this study. Our study found that the age of CM patients, age of migraine onset, headache intensity, BMI, and medication overuse were not associated with the efficacy of the preventive treatment. Therefore, more longitudinal and multicenter clinical studies on CM are needed in the future to clarify the factors affecting the preventive treatment so as to guide clinicians in providing appropriate treatment to CM patients.

According to our follow-ups, 104 patients (14.1%) developed side effects. The most common side effects are dry mouth, drowsiness, and gastrointestinal reactions, but the side effects are not the primary cause of non-compliance for CM patients. The most common reason is appointment difficulty in a headache clinic (31.8%) because headache physicians are still relatively rare compared with other physicians in China. How could the patients get formal and standard treatment if they could not get an appointment at a headache clinic? So strengthening the construction of headache clinics and training more headache physicians are the top priorities for the Chinese headache society.

There are also some limitations in this study. Firstly, this study was conducted in the six first-class hospitals in Chongqing, China, and there is a higher number of patients with severe CM, which also explains why the patients included in this study had a high number of headaches and a long history of migraine. Secondly, this study has a historical retrospective baseline with high recollection bias, and the results may be influenced by many factors.

This study suggests a significantly low compliance rate with CM therapy. The onset of first-time prophylaxis may be related to the number of headache days per month in CM patients, suggesting that prophylaxis for CM should be initiated early. For the Chinese headache society, the best way to increase patient compliance should be treatment at dedicated headache centers and timely visits to headache specialists.

## Data Availability Statement

All datasets generated for this study are included in the article/supplementary material.

## Ethics Statement

Our study was approved by the ethics board at the First Affiliated Hospital of Chongqing Medical University (20151223). All the patients provided written informed consent before enrollment. The trial was performed in accordance with the ethical standards laid down in the 1964 Declaration of Helsinki.

## Author Contributions

GT conceived and oversaw the study. YZ, QL, YL, XL, YY, and WL performed data collection. DY performed data analysis. DY and GT wrote manuscript.

### Conflict of Interest

The authors declare that the research was conducted in the absence of any commercial or financial relationships that could be construed as a potential conflict of interest.
